# Chromatin modification contributes to the expression divergence of three *TaGS2* homoeologs in hexaploid wheat

**DOI:** 10.1038/srep44677

**Published:** 2017-03-16

**Authors:** Wei Zhang, Xiaoli Fan, Yingjie Gao, Lei Liu, Lijing Sun, Qiannan Su, Jie Han, Na Zhang, Fa Cui, Jun Ji, Yiping Tong, Junming Li

**Affiliations:** 1 Center for Agricultural Resources Research, Institute of Genetics and Developmental Biology, Chinese Academy of Sciences, Shijiazhuang 050022, China; 2 State Key Laboratory of Plant Cell and Chromosome Engineering, Chinese Academy of Sciences, Beijing 100101, China; 3 Chengdu Institute of Biology, Chinese Academy of Sciences, Chengdu 610041, China; 4 Hebei Key Laboratory of Molecular and Cellular Biology, Hebei Normal University, Shijiazhuang 050024, China; 5 Institute of Cereal and Oil Crops, Hebei Academy of Agricultural and Forestry Sciences, Shijiazhuang 050035, China; 6 University of Chinese Academy of Sciences, Beijing 100049, China

## Abstract

Plastic glutamine synthetase (GS2) is responsible for ammonium assimilation. The reason that *TaGS2* homoeologs in hexaploid wheat experience different selection pressures in the breeding process remains unclear. *TaGS2* were minimally expressed in roots but predominantly expressed in leaves, and *TaGS2-B* had higher expression than *TaGS2-A* and *TaGS2-D*. ChIP assays revealed that the activation of *TaGS2-B* expression in leaves was correlated with increased H3K4 trimethylation. The transcriptional silencing of *TaGS2* in roots was correlated with greater cytosine methylation and less H3K4 trimethylation. Micrococcal nuclease and DNase I accessibility experiments indicated that the promoter region was more resistant to digestion in roots than leaves, which indicated that the closed nucleosome conformation of the promoter region was important to the transcription initiation for the spatial-temporal expression of *TaGS2*. In contrast, the transcribed regions possess different nuclease accessibilities of three *TaGS2* homoeologs in the same tissue, suggesting that nucleosome conformation of the transcribed region was part of the fine adjustment of *TaGS2* homoeologs. This study provides evidence that histone modification, DNA methylation and nuclease accessibility coordinated the control of the transcription of *TaGS2* homoeologs. Our results provided important evidence that *TaGS2-B* experienced the strongest selection pressures during the breeding process.

Nitrogen, which is a key element for plant growth and reproduction, is an essential building block of nucleic acids and proteins. In plant nitrogen metabolism, glutamine synthetase (GS) is critical for the first step of ammonium assimilation and transformation into glutamine^1^,^2^. In fact, over 95% of the ammonia available to higher plants may be assimilated via the GS/GOGAT pathway^2^,^3^. There are two GS isoforms in higher plants, cytosolic (GS1) and plastic (GS2), which have metabolic functions that do not overlap^4^. In diploid plants, GS1 is usually encoded by three to five genes, which are predominantly expressed in the vascular tissues and are involved in generating glutamine for intercellular N transport^5^. In contrast, diploid species often have a single gene encoding GS2, which is primarily found in chloroplasts and mitochondria^6^,^7^,^8^.

Recently, increasing evidence has demonstrated that GS is crucial for N assimilation and the yield potential of cereal crops, such as rice, wheat and maize^9^,^10^,^11^,^12^. In wheat, favorable haplotypes of *GS2* were found to be associated with improved seedling growth, agronomic performance, and N uptake during vegetative growth or grain N concentration^13^. During study of the mini core collection (MCC) of Chinese wheat and expanded wheat varieties, it was interesting that the three *TaGS2* homoeologs (*TaGS2-A, -B* and *-D*) in hexaploid wheat suffered different degrees of selection during the breeding process. Among the three homoeologs, *TaGS2-B* experienced rigorous selection (one haplotype was eliminated in the breeding process), *TaGS2-A* experienced moderate selection and *TaGS2-D* experienced slight selection^13^.

Bread wheat (*Triticum aestivum* L.) is an allohexaploid grass species (AABBDD genome, 2n = 6x = 42) that arose through hybridization of three related diploid grasses. The role of polyploidy in shaping eukaryotic evolution is particularly evident in flowering plants^14^. Polyploidy may benefit plants by increasing gene expression levels, organ size and flowering time as well as providing a source for novel variants^15^,^16^. Gene multiplication and redundancy result from the combination of several highly similar genomes with three possible evolutionary outcomes for homoeologous genes (homoeologs) in polyploids: retention of the original or a similar function, functional diversification and gene silencing^17^.

Expression divergence of homoeologs is frequently observed in wheat as well as other polyploid plant species^18^,^19^. Epigenetic mechanisms have been found to regulate the expression of homoeologs and organ-specific expression in polyploids. In wheat, only *WLHS1-D* functions in hexaploid wheat out of the three *WLHS1* homoeologs. *WLHS1-A* had a structural alteration in its sequence, and *WLHS1-B* was predominantly silenced by cytosine methylation^19^. For organ-specific expression, all three *TaEXPA1* homoeologs were silenced in seedling roots. In seedling leaves, *TaEXPA1-A* and *TaEXPA1-D* were expressed, but *TaEXPA1-B* was silenced. Transcriptional silencing of three *TaEXPA1* homoeologs in roots was correlated with an increased level of H3K9 dimethylation and decreased levels of H3K4 trimethylation and H3K9 acetylation. Moreover, a higher level of cytosine methylation was detected in the promoter region of *TaEXPA1-B*, which may have contributed to silencing *TaEXPA1-B* in leaves^18^. During cold stress, changes in region-specific histone modification marks promoted alteration of the chromatin structure to facilitate the binding of transcription machinery for *OsDREB1b* expression^20^. Nucleosome conformation has been reported to affect spatio-temporal expression, nucleosome arrangement and hyperacetylation of histones that coordinate control of the high transcription of *PetE* in green shoots and its absence in roots^21^.

Although previous studies have revealed the important function of *GS2* in N assimilation and agronomic performance in wheat^13^, the reason *TaGS2-B* encounters rigorous selection pressures in the breeding process remains unclear. Genetic evidence has indicated that deletion of *TaGS2-B* gene obviously influenced the height and spike number of wheat. The present study indicated that the expression of *TaGS2-B* was higher than *TaGS2-A* and *TaGS2-D* in leaves. Moreover, we demonstrated that epigenetic modifications and nucleosome conformation contributed to the divergent expression of the three *TaGS2* homoeologs and spatial-temporal specific expression of *TaGS2*. Our results clarified the molecular mechanism for divergent expression of *TaGS2* homoeologs, and the high expression level of *TaGS2-B* indicates it has an important function. Therefore, these results provide important information about the rigorous selection pressure for *TaGS2-B* in the breeding process.

## Results

### Identification of *TaGS2* genes in wheat

Full-length cDNA sequences of *TaGS2* homoeologs from the progenitor species of hexaploid wheat (Fig. S1) and two tetraploid wheat samples (Fig. S2) were isolated. We also isolated the full-length cDNA and genomic DNA sequences of three *TaGS2* homoeologs from the winter wheat variety Kn9204. The cDNA sequences of the three *TaGS2* homoeologs were all 1284 bp, with significant conservation among them and only 46 nucleotide substitution sites (Fig. S3). The sequence similarity of the deduced protein sequences was 99.84%. TaGS2-A and -D had identical amino acid sequences and only differed from the TaGS2-B sequence by two amino acids. The greatest variation was observed in the introns of the *TaGS2-A, -B* and *-D* genomic sequences (Fig. S5). Promoter regions (1000 bp regions upstream of the ATG initiation codon) of the three*TaGS2* homoeologs were also isolated for the next experiment (Fig. S4).

**Figure 1:**
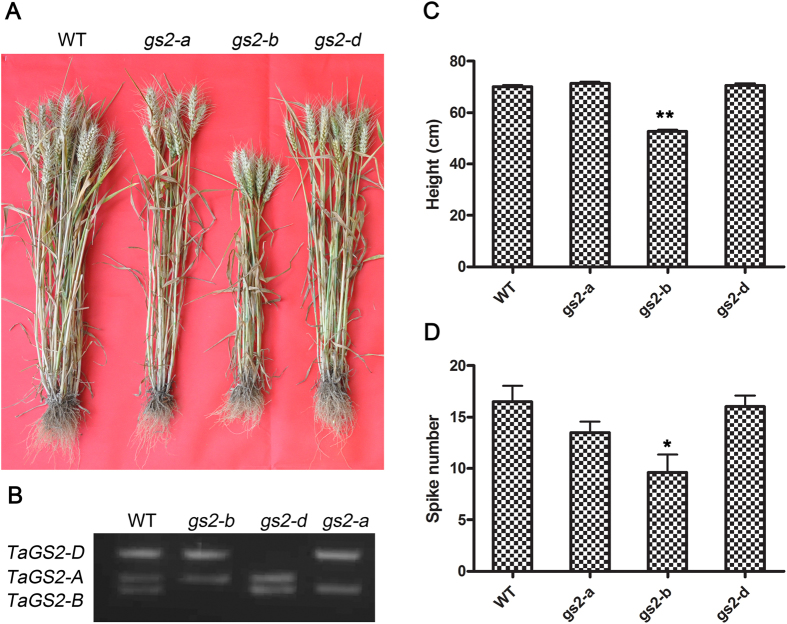
*TaGS2* function is essential for a normal yield in wheat. (**A**) Phenotype of *TaGS2* gene deletion mutants. (**B**) Identification of *TaGS2* gene deletion mutants. (**C**) Plant height. (**D**) Spike number. Error bars indicate ± SE (n = 10). Asterisks indicate the significance of differences between wild-type and *gs2* mutants as determined by the Student’s *t*-test: **P < 0.01, *P < 0.05.

To investigate the function of *TaGS2* in wheat, we screened for *TaGS2* deletion mutants among ion beam-induced mutants of the variety Kn9204 using genomic PCR (Figs 1B and S6). After screening the mutants, the homozygous *gs2-a, b* and *d* mutants were obtained (Fig. 1A). Before further analysis, three homozygous *gs2* mutants were back-crossed twice with their wild-type progenitor Kn9204, and homozygous BC_2_F_3_ mutants were used for phenotype analysis. *TaGS2-B* deficiency severely reduced plant height (Fig. 1C). The average height of mature *gs2-b* plants was nearly 24% lower than wild-type plants. Moreover, deletion of *TaGS2-B* led to a reduced spike number (Fig. 1D).

### Expression patterns of *TaGS2*

**Figure 2:**
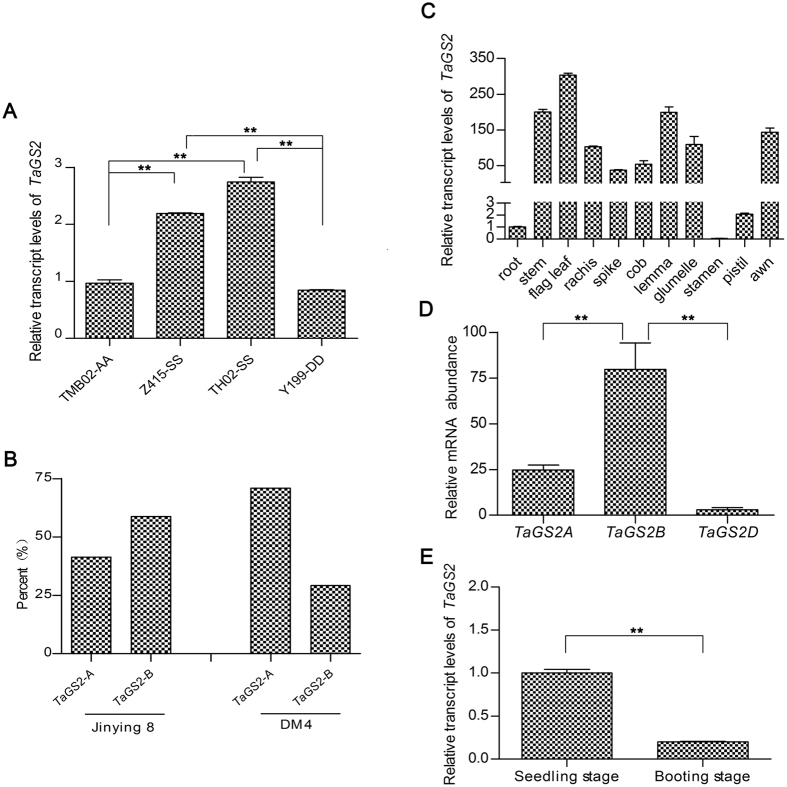
The expression patterns of the *TaGS2* homoeologs. (**A**) Divergent expression of the *TaGS2* homoeologs in the leaves of 7-d-old seedlings of the progenitor species. (**B**) Percentage of *TaGS2-A* and *TaGS2-B* occurring in the leaves of 7-d-old seedlings of tetraploid wheat. (**C**) Tissue-specific expression of *TaGS2* at the booting stage. (**D**) Divergent expression of the three *TaGS2* homoeologs in the leaves of 10-d-old seedlings. (**E**) The expression of *TaGS2* at two developmental stages. For (**A**,**D** and **E**), the data are means ± SD of three independent biological replicates. Asterisks indicate a statistically significant difference (Student’s *t*-test: **P < 0.01).

To analyze the expression of *TaGS2* homoeologs, the progenitor species of hexaploid wheat and two tetraploid wheat samples were examined. The transcription levels of the three *TaGS2* homoeologs differed significantly, and the *TaGS2* transcript levels were higher in the ancestor of the B genome (Z415 and TH02) compared to the progenitor species of the A genome (TMB02) and the donor of the D genome (Y199) (Fig. 2A). Moreover, the occurrence of *TaGS2-B* was 1.42-fold higher than *TaGS2-A* in the tetraploid wheat Jinying 8, but the occurrence of *TaGS2-A* was 2.43-fold higher than *TaGS2-B* in tetraploid wheat DM4 (Fig. 2B). These results suggested that the combination of the highly similar genomes influences the expression of *TaGS2* homoeologs. Next, we studied *TaGS2* homoeologs expression in hexaploid wheat.

The spatial-temporal expression of *TaGS2* was examined with SYBR qRT-PCR in hexaploid wheat Kn9204. The *TaGS2* transcripts were detectable in most tissues at the booting stage, with predominant expressionin the flag leaf and lower transcript levels in roots. *TaGS2* was also expressed in the stem, rachis, spikes, cobs, lemma, glummelle, awn, stamens and pistils at the booting stage (Fig. 2C). To investigate the expression divergence of the three *TaGS2* homoeologs, 10-d-old seedlings were used along with gene-specific primers and probes for TaqMan absolute qRT-PCR analysis (Table S1). The transcript levels of the three *TaGS2* homoeologs differed significantly, with greater *TaGS2-B* levels than *TaGS2-A* and *TaGS2-D* in leaves (Fig. 2D). When different developmental states were considered, the *TaGS2* transcript levels were higher in leaves at the seedling stage than in leaves at the booting stage (Fig. 2E).

### H3K4me3 levels were positively correlated with *TaGS2* transcript abundance

Previous studies have shown that epigenetic changes help maintain homoeologous expression patterns in allopolyploid plants^18^,^19^,^22^,^23^. To gain insight into the histone modification patterns of different organs at various developmental stages, the leaves and roots of 10-d-old seedlings and booting-stage leaves were chosen as starting materials.

**Figure 3:**
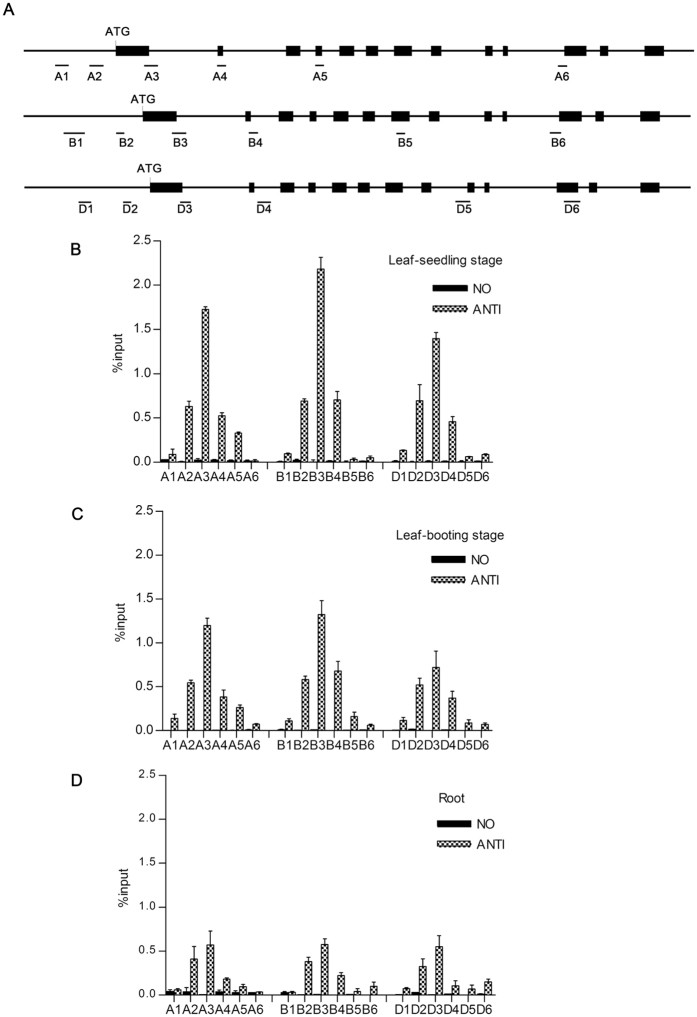
Chromatin immunoprecipitation (ChIP) analysis of the three *TaGS2* homoeologs. (**A**) A diagram showing the three homoeologous *TaGS2* genomic regions with ATG indicating the transcription start site. The black lines denote introns or intergenic regions, and the black rectangles denote coding regions. The regions analyzed for H3K4me3 enrichment in this study are marked by black bars below the diagram. (**B**) Leaves of 10-d-old seedlings. (**C**) Booting-stage leaves. (**D**) Roots of 10-d-old seedlings. (**B**) to (**D**) ChIP to examine the enrichment of H3K4me3 in WT. The data are means ± SD of three independent biological replicates.

Chromatin immunoprecipitation (ChIP) was performed to determine whether H3K4me3 histone modification regulated the divergent expression of the three *TaGS2* homoeologs, and gene-specific primers were designed for *TaGS2-A, TaGS2-B* and *TaGS2-D* for quantitative PCR amplification (Fig. 3A). H3K4me3 levels were high near the start codon for all three *TaGS2* homoeologs, with greater H3K4me3 abundance at these regions in leaves compared to roots. Additionally, enrichment was low at the promoter region located more upstream (Fig. 3B to D). When considering different developmental stages, H3K4me3 enrichment was higher in the leaves of 10-d-old seedlings compared to booting-stage leaves (Fig. 3B,C). Additionally, the H3K4me3 level was higher at the ATG initiation codon region of *TaGS2-B* compared with*TaGS2-A* and *TaGS2-D* (Fig. 3B). Because no differences were found in the known *cis*-elements in the promoter sequences of the three homoeologs, it is likely that the different levels of H3K4me3 contributed to the transcriptional silencing of *TaGS2* in roots and the activation of *TaGS2-B* in leaves. These results indicated that H3K4me3 helped to regulate the divergent expression of the *TaGS2* homoeologs in different organs and developmental stages.

### Negative expression regulation by DNA methylation contributed to the spatial-temporal expression of the three *TaGS2* homoeologs

**Figure 4:**
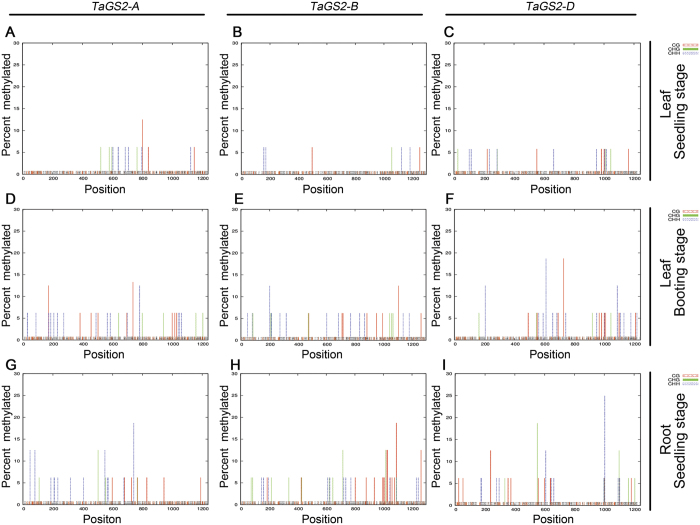
Distribution of methylated cytosine in the 5′ region and exon 1 of the three *TaGS2* homoeologs. (**A**) to (**C**) Leaves of 10-d-old seedlings. **(D**) to (**F**) Booting-stage leaves. **(G)** to **(I)** Roots of 10-d-old seedlings. (**A**,**D**) and (**G**) *TaGS2-A*. (**B**,**E**) and (**H**) *TaGS2-B*. (**C**,**F**) and (**I**) *TaGS2-D*. The methylation status of CG/CHG/CHH sites in the 5′ regions and exon 1 of *TaGS2* homoeologous genes in different tissues and developmental stages. Genomic DNA was treated with sodium bisulfite and used for PCR with primers that amplified fragments in the 5′ regions and exon 1 of the three *TaGS2* homoeologous genes. The sequences were determined in 16 clones of each gene. Sequencing data were analyzed using Kismeth software (http://katahdin.mssm.edu/kismeth/revpage.pl). The colored lines above the x-axis show the percentage of methylation at individual cytosine sites. The short bars at the bottom of the graph show the positions of the cytosines.

Cytosine methylation is another conserved epigenetic marker. In some cases, the amount of methylated DNA is inversely related to the amount of methylated H3K4^24^. In the current analysis of the three *TaGS2* homoeologs, there was an 82% sequence similarity for the 1000 bp region upstream of the ATG initiation codon and the first exon (Fig. S4). Sequence analysis also indicated that the *TaGS2* genes had >50% GC content and contained the criteria for CpG islands. Bisulfite sequencing PCR (BSP) was used to analyze the methylation status of CG/CHG/CHH sites in the 5′ regions and exon 1 of *TaGS2* (Fig. 4). Using the leaves and roots of 10-d-old seedlings and booting-stage leaves as starting materials, the methylation ratio of CG/CHG/CHH sites in the analyzed regions of *TaGS2* was found to be lower in the leaves of 10-d-old seedlings (Fig. 4A to C) compared with booting-stage leaves (Fig. 4D to F). Additionally, a higher methylation ratio and more DNA methylation sites were found in roots compared to leaves (Fig. 4G to I). These data imply that the DNA methylation of the *TaGS2* 5′ regions and exon 1 is associated with a suppressive effect that ultimately affected the tissue- and development-specific transcription of *TaGS2*. A further comparison of the distribution and ratio of methylated cytosines in the 5′ regions and exon 1 showed that *TaGS2-D* (Fig. 4C,F and I) was hypermethylated compared with *TaGS2-A* (Fig. 4A,D and G) and *TaGS2-B* (Fig. 4B,E and H). This finding was consistent with the low expression of *TaGS2-D* in leaves.

### Nuclease accessibility of *TaGS2*

**Figure 5:**
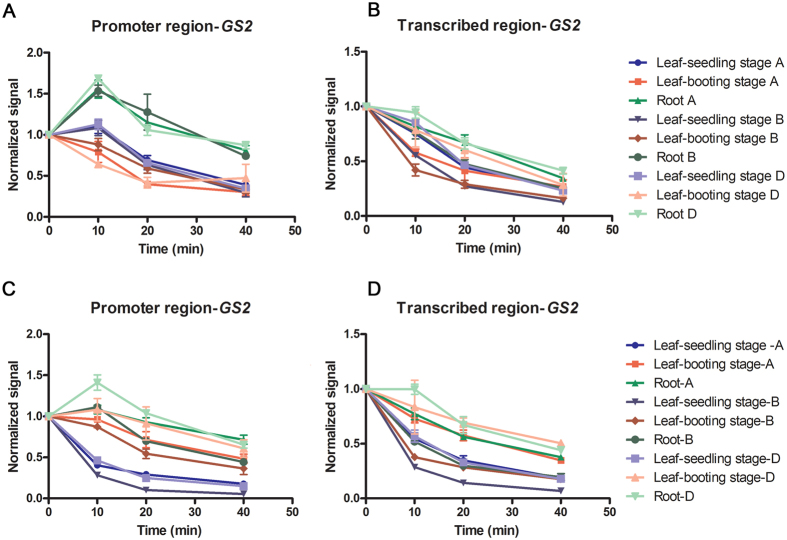
Accessibility changes of three *TaGS2* homoeologs to endonuclease in the roots and leaves. The nuclei were digested with micrococcal nuclease and DNase I for an increasing time period as indicated. The amount of DNA was detected with quantitative PCR using primers specific for three *TaGS2* homoeologs at the promoter and transcribed regions, respectively. Roots and leaves of 10-d-old seedlings and booting-stage leaves were measured. The data are the means ± SD of three independent biological replicates. (**A**) and (**B**) Degradation rates of three *TaGS2* homoeologs with DNase I treatment. (**C**) and (**D**) Degradation rates of three *TaGS2* homoeologs with micrococcal nuclease treatment.

After investigating histone modification and DNA methylation status, we examined the nuclease accessibility of the three *TaGS2* homoeologs at two organs and development stages as a means of assessing the chromatin structure of the genes. We examined the degradation rates of the promoter and transcribed regions of the three *TaGS2* homoeologs in the roots and leaves of 10-d-old seedlings and booting-stage leaves by micrococcal nuclease and DNase I (Fig. 5).

The degradation pattern by both micrococcal nuclease and DNase I was similar in the transcribed region of the three *TaGS2* homoeologs (Fig. 5B and D). For the same *TaGS2* homoeologs, the transcribed region was degraded at a slower rate in roots than in leaves, especially leaves of 10-d-old seedlings. In contrast, the degradation rates of the transcribed region of GAPDH showed no difference between roots and leaves (Fig. S8). This finding indicated that the chromatin had a closer conformation in roots compared with leaves for *TaGS2* homoeologs. In the same tissues, the transcribed regions of *TaGS2-A* and *TaGS2-D* were degraded slower than *TaGS2-B*. It was suggested that *TaGS2-D* and *TaGS2-A* possessed a relatively close conformation compared with*TaGS2-B*. Overall, the most obvious difference in the chromatin structure was between *TaGS2-D* in roots and *TaGS2-B* in leaves (Fig. 5B and D).

Comparing the digestion experiments of the *TaGS2* promoter region by micrococcal nuclease and DNase I had consistent results (Fig. 5A and C). In contrast to the transcribed region, the digestion rates of the*TaGS2* promoter region were slow (Fig. 5). In other words, the *TaGS2* promoter region processed a relatively closer conformation than the transcribed region. The promoter sequence was degraded rapidly in the leaves of two developmental stages. Whereas no obvious digestion by micrococcal nuclease and DNase I was observed in roots (Fig. 5A and C). The relative inaccessibility of the promoter region was correlated with the inactive state of *TaGS2* in roots. The positioning of nucleosomes on promoters inhibited transcription because it occluded access both the transcription factors and the transcriptional machinery at their cognate sites. Thus, a silent state of *TaGS2* in roots and active transcription of *TaGS2* in leaves was demonstrated (Fig. 5A and C).

## Discussion

Polyploidy is recognized as a major force in the evolution of flowering plants, and many important crop plants, such as common wheat, have the hallmarks of allopolyploidy. Polyploidy leading to divergent expression of transcriptional genes has been widely confirmed in the allotetraploid *Arabidopsis*^25^,^26^ and cotton^27^. These reports also indicated that silencing was achieved through epigenetic rather than genetic modifications^18^,^19^,^22^. For the three *WLHS1* homoeologs, *WLHS1-A* had no function with an insertion, and *WLHS1-B* was predominantly silenced by cytosine methylation^19^. Recently, an analysis of the three *TaEXPA1* homoeologous genes in hexaploid wheat demonstrated the tissue-specific expression of *TaEXPA1* homoeologs was caused by epigenetic regulation^18^. The available data suggested that various mechanisms affected regulation of the expression and the fate of homoeologous genes, including altered gene structures and epigenetic modifications.

Hexaploid wheat has evolved from two spontaneous hybridization events. The expression of *TaGS2* homoeologs was divergent in diploid, polyploid and cultivated hexaploid wheat (Fig. 2A,B and D). Bread wheat (AABBDD, 2n = 42) is one of the most important food crops and *TaGS2* is a key gene involved in nitrogen assimilation. Thus, it is meaningful for us to investigate how the expression divergence of the *TaGS2* homoeologs was regulated in hexaploid wheat. We found that three *TaGS2* homoeologs had a similar genomic structure in the coding region with no apparent insertions or deletions detected. Using ChIP analysis, we found that higher levels of *TaGS2-B* (compared to *TaGS2-A* and *TaGS2-D*) were associated with increased H3K4me3 abundance (Fig. 3). Methylation of H3K9 and H3K27 are known as heterochromatic marks and are related to gene repression^28^. However, we did not find different enrichment of H3K9me2 between the three *TaGS2* homoeologs or different tissues (Fig. S7), and no enrichment of H3K27me3 was detected in the leaves and roots of 10-d-old seedlings (data not shown). We further examined the methylation levels of the 5′ regions and exon 1 of the three *TaGS2* homoeologs using BSP. Gene-specific hypermethylation was detected at *TaGS2-D*, which was consistent with the low expression of this gene (Fig. 4).

*TaGS2* transcript levels were higher at the seedling stage, especially in the leaves, which was followed by the booting stage. Additionally, minimal expression was detected in the roots and reproductive organs, such as the stamens and pistils (Fig. 2A). Epigenetic modifications have been correlated with tissue-specific expression in plants^24^,^29^,^30^. Similarly, our ChIP and BSP analyses demonstrated a positive correlation between H3K4me3 and *TaGS2* expression as well as a negative correlation between DNA methylation and tissue-specific expression and divergent expression at different developmental stages. These results were consistent with the hypothesis that epigenetic modifications helped to mark developmental states.

Nucleosomes positioned over *cis*-elements inhibited transcription by preventing transcription factors from accessing their binding sites^31^. Endonucleases possess different DNA digestion characteristics and exhibit different sequence specificities. Micrococcal nuclease preferentially attacks the internucleosomal linker regions but not the DNA sequences that were wrapped around the nucleosome. In contrast, DnaseI cleaves the DNA wrapped around the nucleosome at positions where the minor groove faced away from the nucleosome^32^. Because the digestion of DNA by both enzymes was influenced by the presence of nucleosomes, the digestion data for both enzymes should complement each other and reflect the nucleosome structure along three *TaGS2* homoeologs.

The promoter region of three *TaGS2* homoeologs were less accessible to digestion in roots than the same region in leaves (Fig. 5A and C), which suggested that these promoters possesses a closed conformation in roots, and this closed conformation was also correlated with the lack of basal transcription and was likely to suppress transcription in roots by preventing the binding of transcription factors. The silencing of the pea plastocyanin gene (*PetE*) in roots^21^ and closed chromatin conformation at the promoter region suppressed the transcription of *OsDREB1b* gene^20^, which was consistent with our observations. Changes in chromatin structure have been studied for many plant genes, such as *Adh* in Arabidopsis^33^, tomato proteinase inhibitor-1^34^, Chlamydomonas *HSP70A* and the *RbcS2* gene during transcription activation^35^. Wheat is an allohexaploid species, and the promoter regions of the three *TaGS2* homoeologs possess similar nuclease accessibility for the same tissues, which suggests that the chromatin structure of a promoter region is important to transcription initiation for spatial-temporal reasons rather than reasons specific to *TaGS2* homoelogous genes.

The chromatin conformation of the transcribed region of the three *TaGS2* homoeologs and its effects on transcription was also examined in roots and leaves. In contrast to the promoter region, the transcribed regions possessed different nuclease accessibility among the three *TaGS2* homoeologs in the same tissues. It is suggested that the chromatin structure of the transcribed region participated in the fine adjustment of *TaGS2* homoeologs (Fig. 5B and D).

According to the ChIP, BSP and nuclease accessibility experiments, we demonstrated that the chromatin structure contributed to the expression divergence of three *TaGS2* homoeologs during wheat development. Moreover, this study provides evidence that the three processes coordinated control of the transcription of *TaGS2* homoeologs. Our results clarified the molecular mechanisms for divergent expression of *TaGS2* homoeologs, and the high expression level of *TaGS2-B* indicates that it has an important function. Therefore, these results provide important information about the stronger selection pressures encountered by *TaGS2-B* compared with *TaGS2-A* and *TaGS2-D* in the breeding process.

## Methods

### Plant materials and growth conditions

The progenitor species of hexaploid wheat were used to analyze the expression of *TaGS2* homoeologs, including the diploid wild einkorn wheat TMB02 (*T. urartu*, AA, 2n = 14), two ancestors of the B genome Z415 and TH02 (*Ae. speltoides*, SS, 2n = 14) and the donor of the Dgenome Y199 (*Ae. tauschii*, DD, 2n = 14). The tetraploid wheat DM4 (*T. dicoccum*, AABB, 2n = 28) and durum wheat Jinying 8 (*T.durum*, AABB, 2n = 28) were also used in our experiments.

The hexaploid wheat cultivar Kn9204 (wild-type) was used for experiments. Field trials were carried out at Luancheng Agro-Ecosystem Experimental Station, CAS, China, in 2014. Twenty-five seeds per line were individually sown by hand in 2 m rows that were 25 cm apart. In the laboratory, plants were grown in a phytotron at 25 °C with a 16 h light/8 h dark cycle, and 10-d-old seedlings were used for the following analyses.

### Cloning of *TaGS2* genes

All the PCR primers used in this study are listed in Table S1.

The full-length genomic DNA of the *TaGS2* homoeologous genes were obtained by PCR-directed cloning based on the *TaGS2* sequences in NCBI GenBank (GQ169685, GQ169687 and GQ169688^13^). The promoter regions of the *TaGS2* homoeologs were cloned using the primer pairs GS2-A016 F1/GS2-A016 R1 for *TaGS2-A*, GS2-B017 F1/GS2-B017 R1 for *TaGS2-B* and GS2-D018 F2/GS2-D018 R1 for *TaGS2-D*. The downstream regions were cloned using WLF1-A/F1spAR1-A, F1spAF1-A/GS2R6, GS2F5/GS2R4 and GS2F2/WLR1 for *TaGS2-A*; WLF-B/GS2R6, GS2F5/GS2R4 and GS2F2/WLR1 for *TaGS2-B*; and WLF-D/GS2R6, GS2F5/GS2R4 and GS2F2/WLR1 for *TaGS2-D*. These fragments were then assembled to obtain the full-length sequences of the three *TaGS2* genes in Kn9204.

### Gene expression analyses

Total RNA from plant tissues was extracted using TRIzol reagent (Invitrogen). The first-strand cDNA was synthesized from DNase I-treated total RNA using the PrimeScript RT Reagent Kit (TaKaRa) according to the manufacturer’s instructions.

For the expression of *TaGS2* homoeologs in tetraploid wheat, leaves of 7-d-old seedlings were collected for RNA extraction. RT-PCR products were purified and cloned into the T-vector. About fifty clones of each tetraploid wheat material were sequenced to distinguish between *TaGS2-A* and *TaGS2-B*. The occurrence of each *TaGS2* homoeolog transcript was determined according to the number of corresponding clones. The primers used for RT-PCR were 5′-ATGGCGCAGGCGGTGGTG-3′ and 5′-TCATACCTTCAGCGCCAGCTTCTTG-3′.

For TaqMan absolute quantification real-time RT-PCR analysis, leaves of 10-d-old seedlings were collected for RNA extraction, and the cDNA sequences from the three *TaGS2* genes were cloned into pEASY-T according to the GenBank data (GQ169685, GQ169687 andGQ169688), which corresponded to *TaGS2-A, -B* and *-D*. Plasmid DNA was used to prepare absolute standards. A260 readings were used for the concentration measurements and converted to the number of copies using the molecular weight of the DNA. Quantitative PCR was performed using the Applied Biosystems 7500 real-time PCR system with TaqMan gene expression probes for *TaGS2-A*, -*B* and *-D*. The wheat *β-Tubulin* gene (U76745.1) was used as an internal reference. Each experiment was repeated twice with independent samples. The primer sequences that were used are listed in Table S1.

Relative quantification real-time RT-PCR analysis was performed in triplicate using the ABI7500 system and the SYBR RT-PCR kit (TaKaRa). Leaves of 10-d-old seedlings and tissues at the booting stage were collected for RNA extraction and qRT-PCR assays. The relative transcript level was calculated from three replicates using the comparative Ct method after normalization to the *β-Tubulin* control. The primer sequences that were used are listed in Table S1.

### ChIP

ChIP was performed as previously described^36^. Leaf tissue was completely ground in liquid nitrogen and cross-linked in 1% formaldehyde (Sigma-Aldrich) for 10 min on ice. The chromatin was pelleted by centrifugation and sonicated into ~500 bp DNA fragments. The lysate was pre-cleared with 50 mL protein-A agarose beads/salmon sperm DNA (Millipore) for 1 h, then incubated with anti-H3K4me3 (Abcam) antibodies overnight. The anti-H3K4me3 antibodies were used at 5 mg per 0.5 g of leaf tissue. The bound chromatin was purified using columns from the Qiagen plasmid extraction kit. Real-time PCR was conducted in triplicate on the input with no antibody control and antibody-bound DNA. Homoeologous gene-specific primers were designed as follows: qPCR-A-F1/qPCR-A-R1, qPCR-A-F2/qPCR-A-R2, qPCR-A-F3/qPCR-A-R3, qPCR-A-F4/qPCR-A-R4, qPCR-A-F5/qPCR-A-R5, qPCR-A-F6/qPCR-A-R6; qPCR-B-F1/qPCR-B-R1, qPCR-B-F2/qPCR-B-R2, qPCR-B-F3/qPCR-B-R3, qPCR-B-F4/qPCR-B-R4, qPCR-B-F5/qPCR-B-R5, qPCR-B-F6/qPCR-B-R6; qPCR-D-F1/qPCR-D-R1, qPCR-D-F2/qPCR-D-R2, qPCR-D-F3/qPCR-D-R3, qPCR-D-F4/qPCR-D-R4, qPCR-D-F5/qPCR-D-R5, qPCR-D-F6/qPCR-D-R6 (Table S1). Three biological replicates were conducted for each sample to ensure reproducibility.

### Bisulfite sequencing

Genomic DNA was isolated from the leaves and roots of 10-d-old seedlings and from the booting-stage flag leaves using the DNeasy Plant Mini Kit (Qiagen). The EZ DNA Methylation-Gold Kit was used for bisulfite treatment of genomic DNA according to the manufacturer’s instructions (ZYMO Research). The bisulfite-treated DNA was used for PCR amplification, and the clones were randomly sequenced. The primer sets were designed using Methyl Primer Express Software V1.0 (Applied Biosystems). Homoeologous gene-specific primers were designed as follows: BSP-A-F1/BSP-A-R1, BSP-A-F2/BSP-A-R2, BSP-A-F3/BSP-A-R3, BSP-A-F4/BSP-A-R4; BSP-B-F1/BSP-B-R1, BSP-B-F2/BSP-B-R2, BSP-B-F3/BSP-B-R3, BSP-B-F4/BSP-B-R4; BSP-D-F1/BSP-D-R1, BSP-D-F2/BSP-D-R2, BSP-D-F3/BSP-D-R3, BSP-D-F4/BSP-D-R4, BSP-D-F5/BSP-D-R5 (Table S1). The PCR products were cloned into the pGEM-T plasmid vector (Promega) and sequenced using vector-specific primers. These fragments were subsequently assembled to obtain the 5′ region sequences of the three *TaGS2* genes. Sequencing data were analyzed using Kismeth software^37^, http://katahdin.mssm.edu/kismeth/revpage.pl).

### Enzyme accessibility assay

Enzyme (MNase and DNase I) accessibility assay to monitor chromatin remodelling in three *TaGS2* homoeologs was done as described by Chua and Brown^21^. Nuclei from 0.125 g roots and leaves of 10-d-old and booting-stage seedlings were digested with MNase (15 U/ml) and DNase I (2.5 U/ml) for 0, 10, 20 and 40 min. Three replicates of each nuclease treatment were performed and analyzed by quantitative PCR. The specific primers of the *TaGS2* homoeologs promoter region were designed as BSP-A-F1/BSP-A-R1, BSP-B-F1/BSP-B-R1, and BSP-D-F1/BSP-D-R1. The specific primers of the *TaGS2* homoeologs transcribed region were designed as BSP-A-F6/BSP-A-R6, BSP-B-F6/BSP-B-R6, and BSP-D-F6/BSP-D-R6. The amount of PCR product obtained for the different digestion time points was normalized with the amount of product present at the zero time point.

## Additional Information

**How to cite this article:** Zhang, W. *et al*. Chromatin modification contributes to the expression divergence of three *TaGS2* homoeologs in hexaploid wheat. *Sci. Rep.* **7**, 44677; doi: 10.1038/srep44677 (2017).

**Publisher's note:** Springer Nature remains neutral with regard to jurisdictional claims in published maps and institutional affiliations.

## Supplementary Material

Supplementary Data
