# Supplementary material for: Chromatin modification contributes to the expression divergence of three TaGS2 homoeologs in hexaploid wheat
Source: Sci Rep. 2017 Mar 16;7:44677. doi: 10.1038/srep44677 (PMC5353557; doi:10.1038/srep44677)
Supplement: Supplementary Data [file srep44677-s1.pdf]

Chromatin modification contributes to the expression divergence of three *TaGS2* homoeologs in hexaploid wheat

Wei Zhang [a,b,1], Xiaoli Fan [d,1], Yingjie Gao [c,1], Lei Liu [a,b], Lijing Sun [e], Qiannan Su [a,f], Jie Han [a,f], Na Zhang [a,f], Fa Cui [a], Jun Ji [a,b], Yiping Tong [b], Junming Li [a,b,2]

[a] Center for Agricultural Resources Research, Institute of Genetics and Developmental Biology, Chinese Academy of Sciences, Shijiazhuang 050022, China

[b] State Key Laboratory of Plant Cell and Chromosome Engineering, Chinese Academy of Sciences, Beijing 100101, China

[c] Hebei Key Laboratory of Molecular and Cellular Biology, Hebei Normal University, Shijiazhuang 050024, China

[d] Chengdu Institute of Biology, Chinese Academy of Sciences, Chengdu 610041, China

[e] Institute of Cereal and Oil Crops, Hebei Academy of Agricultural and Forestry Sciences, Shijiazhuang 050035, China

[f] University of Chinese Academy of Sciences, Beijing 100049, China

[1] These authors contributed equally to this work.

[2] Address correspondence to ljm@sjziam.ac.cn.

Tel: +86-311-85887272

Fax: +86-311-85815093

Supplementary Data

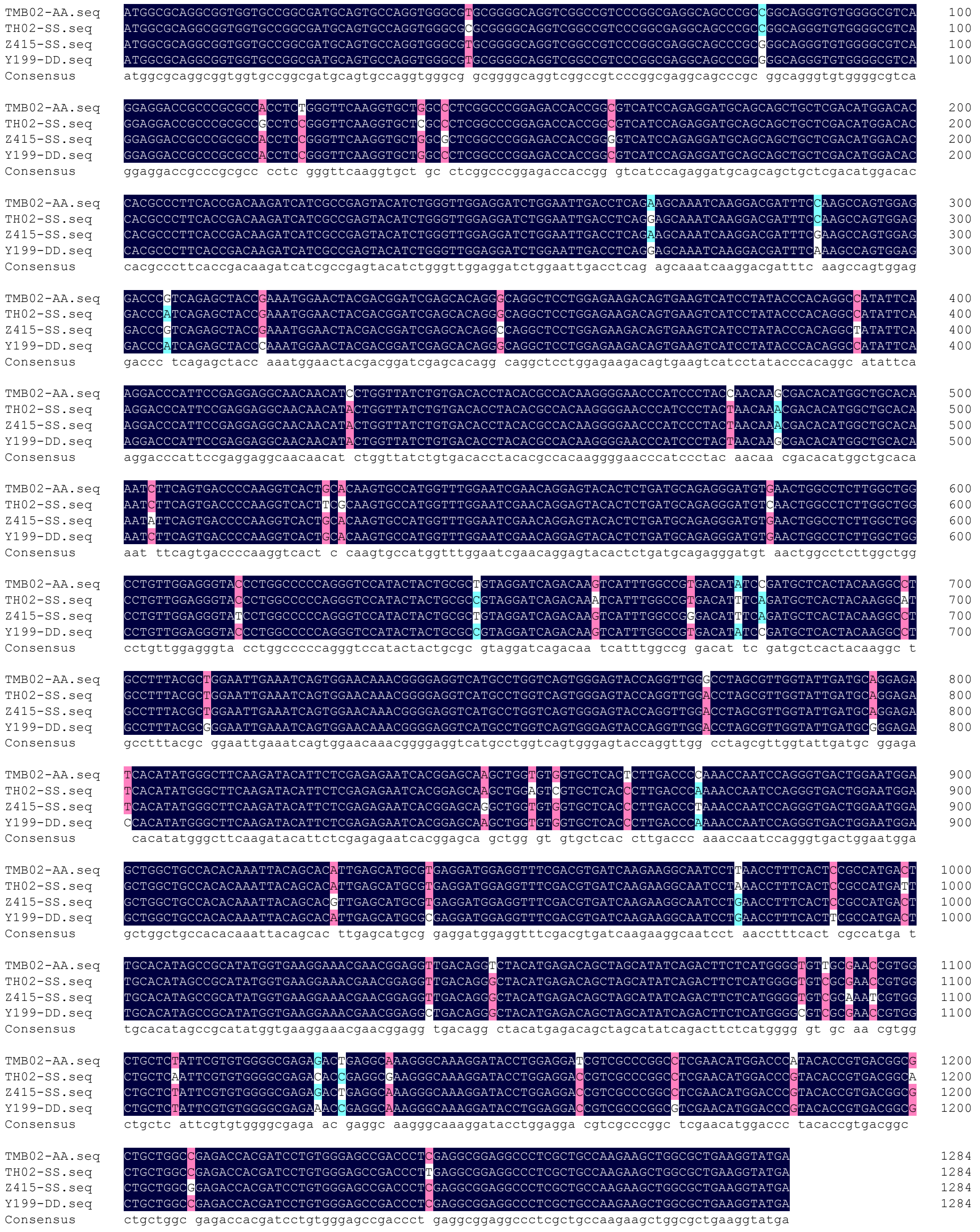

Fig S1. cDNA sequences alignment of *TaGS2* homoeologs in the progenitor species .

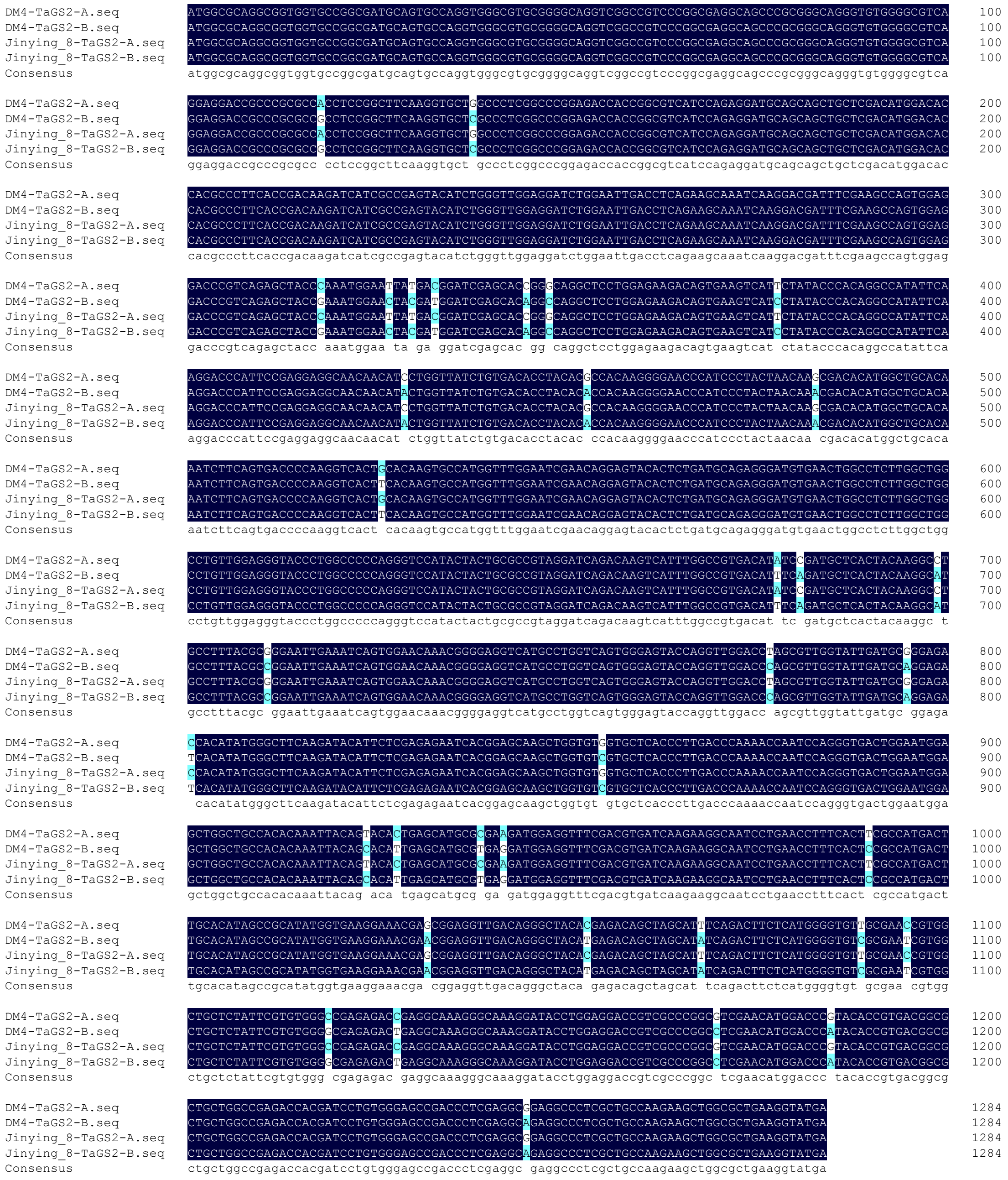

Fig S2. cDNA sequences alignment of *TaGS2-A* and *TaGS2-B* in tetraploid wheat.

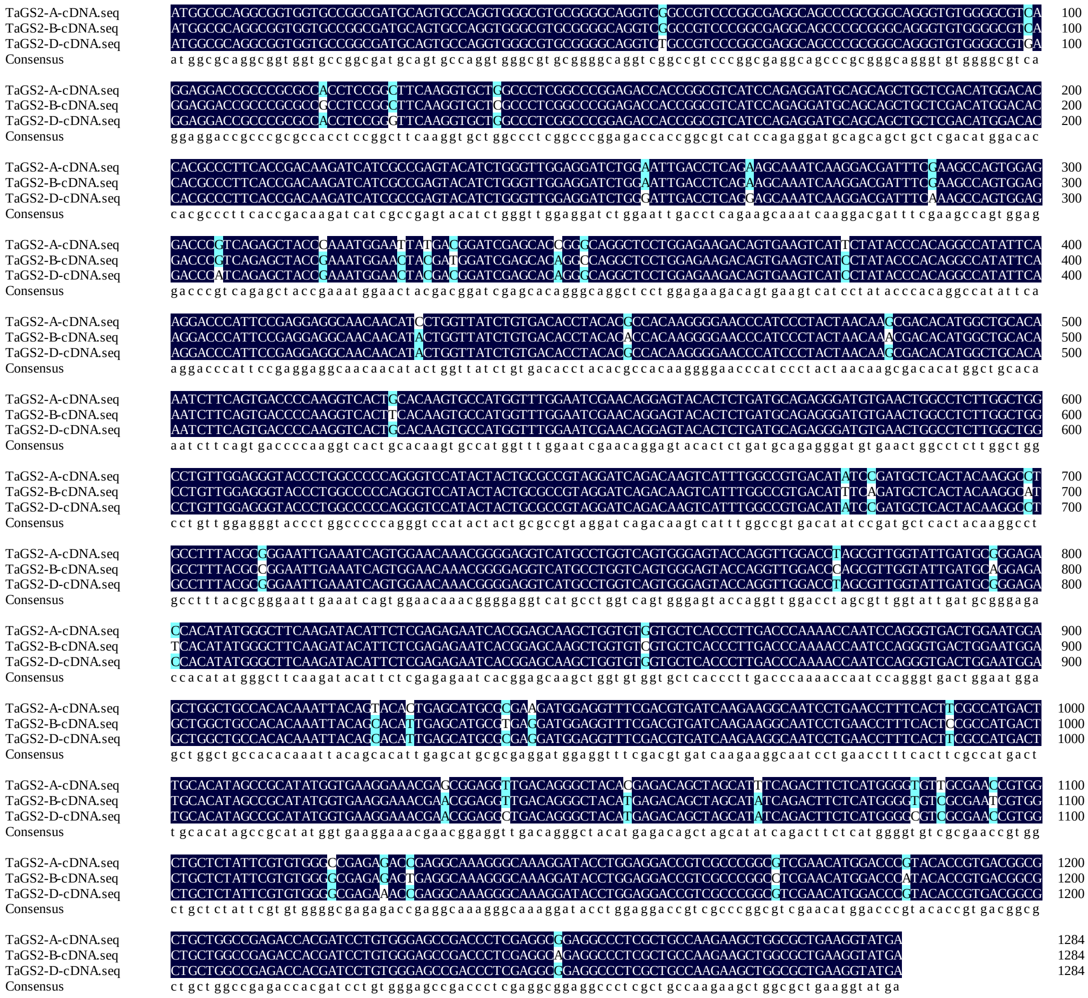

Fig S3. Sequence alignment of the cDNA sequences of *TaGS2-A*, *TaGS2-B* and *TaGS2-D* in hexaploid wheat Kn9204.

```
TaGS2-A-promoter-1000.seq   CGTGCAACCTGGAGTAGTTTGTGGTTTCCACTTTTGCCGTTCTACGACGGAGGATAAACTCGCCTGCTAGGCTACTAGTG   80
TaGS2-B-promoter-1000.seq   CAGGCGAATTCCTGTAGCGCATGCACTGTAGTCGGGTCACTGTA. GCCGGATTCCTGTAGCATAATCCCTGTAGCATATC   79
TaGS2-D-promoter-1000.seq   CTTGCAACCTGGAGTAGTTTGCGGTTTCCACTTTTGCCGTGCTACGACGTAGGATAAAACCAC. . . . . . . . CTACTAGTG   72

TaGS2-A-promoter-1000.seq   TGGTTGCACTTGCACAAGAAGGCAGGGCCAAAAGTACAGGCAGCCCATGTGTGCTACCGCTGCTGCTGGGCGCTGTCACA   160
TaGS2-B-promoter-1000.seq   CTGTAGCTCATACACTGTACCTTTCAGCTGTTCACACG. . . AATCTGTCCATAGATTTTTGATTAGACGGTGTGGAGAGA   156
TaGS2-D-promoter-1000.seq   TGGTTGCACTTGCACAAGAACGCAGGGCCAAAAGTAGAGGCAGCCCATGTGTG. . . . . . CTGCTGCTGGGCGCTGTCACA   146

TaGS2-A-promoter-1000.seq   GTGTCACTGCTAGTCCGTCCGTCCCCGGCACCGGGCAGCCCGACTGGAAGTGGACCAAGCTAGGGTGCCCCCATGACAGG   240
TaGS2-B-promoter-1000.seq   GGAGCAAGGC. . ATCATACTGTTCAC. . CTGTGACTCGCTC. ACGGCAAGTC. ACCGGATCTCAGTCCCCCCATGACAGG   230
TaGS2-D-promoter-1000.seq   GTGTCACTGCTAGAGTGCCCGTCCCCGGCACCGGGCAGCCCGACTGGAAGTGGACCAAGCTAGGGTGCCCCCGTGACAGG   226

TaGS2-A-promoter-1000.seq   CCACCCGGCGCAAAGA. CATGCTGCACCTGACAAAGGGG. . . . . . . . . . . . . . . . . . . . . . . TCCATCCATCCATCATCC   296
TaGS2-B-promoter-1000.seq   CCACCCGACCCAAAAAACACGCTGCACCTGACAAAGGGGATGAAGGGATGACAGTCCGTCCGTCCATCCATCCATCATCC   310
TaGS2-D-promoter-1000.seq   CCACCCGACGCAAAGA. CATGCTACACCTGACAAAGGGGATGAAGGGATGACTGTCCGTCCATCCATCCATCCATCATCC   305

TaGS2-A-promoter-1000.seq   TATACGGCTACACCCCCAATTATTGGCCCCGGGGTCAATGAGACACGCACGCCGCACAGCACATAGTTTGCTTGCGTTGC   376
TaGS2-B-promoter-1000.seq   TATACGGCTACACCCCCAATTATTGTCCCCGGGGTCAATGAGACAC. . . . GCCGCACAGCACATAGTTTGCTTGCGTTGC   386
TaGS2-D-promoter-1000.seq   TATACGGCTACACCCCCAATTATTGGCCCCGGGGTCAATGAGACAC. . . . GCCGCCCAGCACATAGTTTGCTTGCGTTGC   381

TaGS2-A-promoter-1000.seq   GGTGCAGTGCAGGTCTTGCTCCGCTCTGATGCATCTGGCTGGGTTTGGTTGGAGAGGTCGCTTGG. . . . GCTGGG. . . . G   448
TaGS2-B-promoter-1000.seq   GTTGCGGTGCAGGTCTTGCTCCGCTCTGATGCATCTGGCTGGGTTTGGTTGGAGAGGTCGCCTGGCTGGGCTGGGTATGG   466
TaGS2-D-promoter-1000.seq   G. . . . . GTGCAGGTCTTGCTCCGCTCTGCTGCATCTGGCTGGGTTTGGTTGGAGAGGTCGCCTGCCTGGGCTGGGTATGG   456

TaGS2-A-promoter-1000.seq   CGGGAGACGGACGTGACAAGGAGAGGCAGCCGCATCTTTCATTTCATATCCGGAGCCAG. . . AGGGAAGGAACA. . . . . .   519
TaGS2-B-promoter-1000.seq   CGGGAGGCGGACGTGACAAGGAGAGGCAGCCGCATCTTTCATTTCATATCCGGAGCCGGGGAGGGAAGGAACAAGGAAC   546
TaGS2-D-promoter-1000.seq   CGGGAGGCGGACGTGACAAGGAGAGGCAGCCGCATCTTTCATTTCATATCCGGAGAGAAG. . GAACAAGGAACACA. . . .   530

TaGS2-A-promoter-1000.seq   . . . TTGTAAACTCTATCCGAGATGTGTGTGCCTTCTTTGGGCAGACGGCGCTTGGTTTCTGGACCGGTTTGCCTCCGGTG   596
TaGS2-B-promoter-1000.seq   ATATTGTAAACTCTATCCGAGATGTGTGTGCCTTCTTTGGAC. . . . . . . . . . . GGTTT. . . . . . . . . TTTGCCTCCGGTG   606
TaGS2-D-promoter-1000.seq   TTGTAAACAACTCTATCCGAGATGTGTGTGCATTCTGGGCGGGG. . . . . . . . CAGTCG. . . . . . . . . . TTGCCTCCGGTG   592

TaGS2-A-promoter-1000.seq   CGGCTGCAAGGGGCGCCTCAAATCTCGTCCACCCAGAGCCCTATCGCCTAATCAAGAGCCAGATGCCACTGCCCGCGCCG   676
TaGS2-B-promoter-1000.seq   CAGCTGCAAGGGCCGCCTCAAATCTCGTCCACCCAGAGCCCTATCGCCTAATCAAGAGCCAGATGCCACTGCCCGCGCCG   686
TaGS2-D-promoter-1000.seq   CAGCTGCAAGGGGCGCCTCAAATCTCGTCCACCCAGAGCCCTATCGCCTAATCAAGAGCCAGATGCCACTGCCCGCGCCG   672

TaGS2-A-promoter-1000.seq   CGGCCACTAGAATCTCCCCCGCAATCTAAATTACTTGTACCACCGGCCTCCGCTCCCATAAATATAAATAATATTTCCAG   756
TaGS2-B-promoter-1000.seq   CGGCCACTAGAATCTCCCCCGCAATCTAAATTACTTATACCACCGGCCTCCGCTTCCAT. . . . . . AAATAATATTTCCAG   760
TaGS2-D-promoter-1000.seq   CGGCCACTAGAATCTCGCCCGCAATCTAAAATACTTATAC. GCTTCCTTCCTTCAAAACCACATAGATAAATATTTCCAG   751

TaGS2-A-promoter-1000.seq   CGCGACCTCGCAAGGTCGC. . . . CTCCCCTTCCCTCCATCCTCTCCCCCCTCGTCTCGTCCGCGTGCTTGTCCGTCTCTC   832
TaGS2-B-promoter-1000.seq   CGCGATCTCGCAAGGTCGTTCCGCCCCCCTTCCCTCCCTCCTCTCCTCCCTCGTCTCGTCCCCGTCGTTG. . . . TCTCTC   836
TaGS2-D-promoter-1000.seq   CGCGATCTCGCA. GGTCGCCCC. GGCCCCTTCCCTCCGTCCCCTCCTCCCTCGTCTCGTCCGCGTCCTTG. . . . . . . . TC   821

TaGS2-A-promoter-1000.seq   TGGTGAGTGCCGCGGACTCCTCGATGCAATGCTTCTTGGAGCACCCGGCTTCTTCAATCTCTGTTGCGTTCATTCGCTGA   912
TaGS2-B-promoter-1000.seq   CGGTGAGTACTGCGGACTCCTCGATGCAATGCTTGTTCGTTTCTCCGTTGCATTCGCCGCCGGCGAGCGATCGGCGCTAA   916
TaGS2-D-promoter-1000.seq   AGGTGAGTTCTACGGACTTCTCGATGCAATGCTTCCTCGATTCTCTGTTGCATTCGCCGCCGGCGGTTGATCGGCGGCAA   901

TaGS2-A-promoter-1000.seq   TCAACCGCAATGGCGCTGCTTTGGTTTGGTTGATCAGC. . . . . . . . . TAGCGCTGAAGGG. . GAAGGCGGCGGCCG. . . GA   978
TaGS2-B-promoter-1000.seq   T. GGCGCTGTTTGCTTTTCTGTGCTTTGGTTGATCAGC. . . . . . . . . TAGCGCTGAAGGG. . GAAGGCGGCGGAGT. . AA   982
TaGS2-D-promoter-1000.seq   T. GGCCCTGTTTCCTTTGCTTTGCTCCGGCTGATCATCAGTTATGTCTCTTGCTGAAGGTTTGGAGGCGGCAGAGTACCA   980

TaGS2-A-promoter-1000.seq   GTAAGTAAGTAAGCAGCGGC   998
TaGS2-B-promoter-1000.seq   GTAAGTAAGCAAGCAGCGGC   1002
TaGS2-D-promoter-1000.seq   GTAAGTAAGTAAGTAGCGGC   1000
```

Fig S4. Sequence alignment of the promoter sequences of *TaGS2-A, TaGS2-B* and *TaGS2-D* in hexaploid wheat Kn9204.

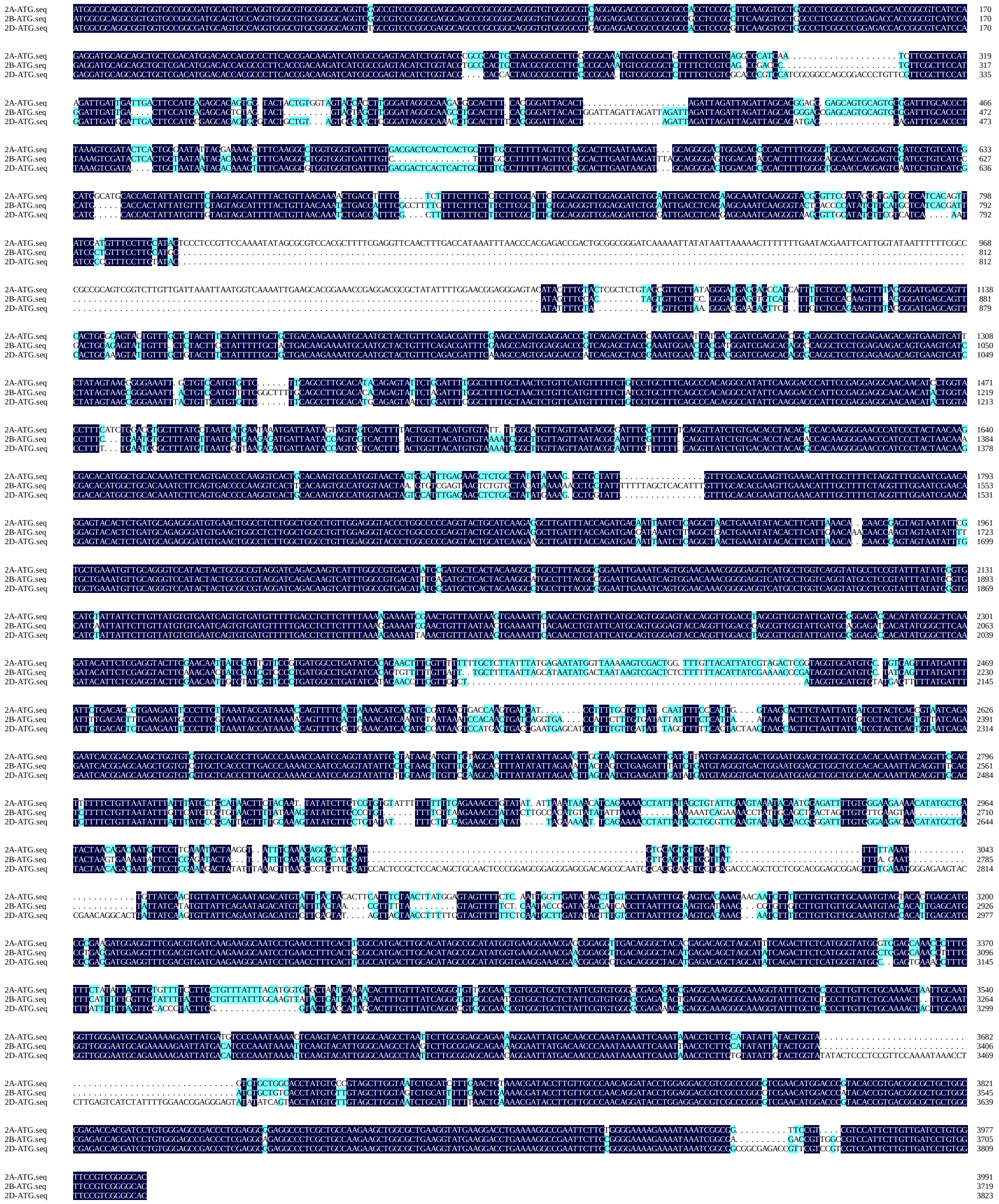

Fig S5. Sequence alignment of the DNA sequences of *TaGS2-A, TaGS2-B* and *TaGS2-D* in hexaploid wheat Kn9204.

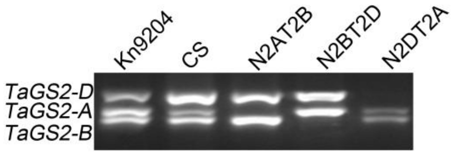

Fig S6. Chromosome mapping of the three *TaGS2* homoeologous genes in Chinese Spring (CS) nulli-tetrasomic lines.The line names specify which chromosomes are present (e.g., N2AT2B is nulli-2A tetra-2B). Kn9204, wild-type plant. The genomic origin of the PCR products is indicated on the left.

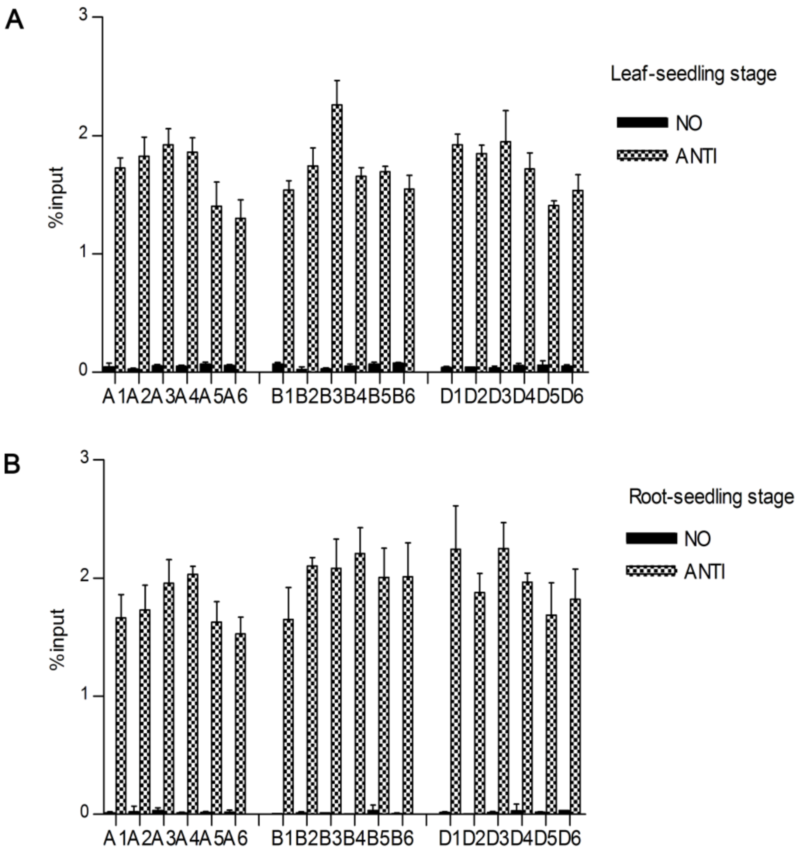

Fig S7. ChIP with anti-H3K9me2 antibodies to determine the levels of H3K9me3 at *TaGS2* in WT.

(A) Leaf of 10-d-old seedling.

(B) Root of 10-d-old seedling.

A

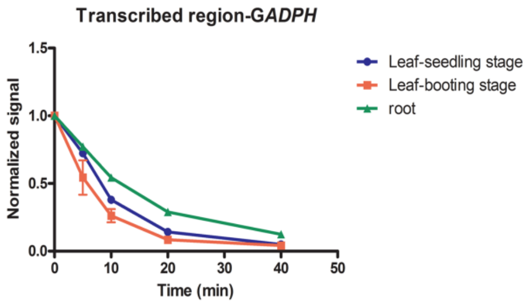

B

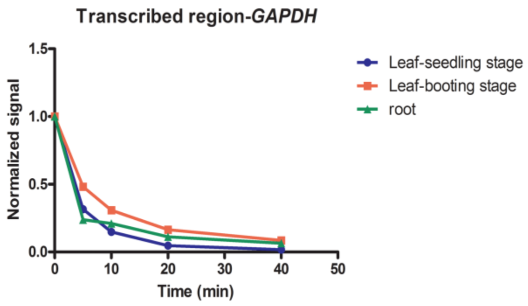

Fig S8. Accessibility of transcribed region of *GAPDH*.

(A) Accessibility of transcribed regions to Micrococcal Nuclease.

(B) Accessibility of transcribed regions to Dnase I.

Table S1. Primers used for PCR, RT-PCR and real-time RT-PCR analysis.

**Experiment**	**Name**	**Primer sequence (5'-3')**
genomic PCR		
	WLF-B	TTCCCTCCCTCCTCTCCTC
	WLF-D	TTCCCTCCGTCCCCTCCTC
	WLF1-A	GCGGAGTAAGTAAGTAAGCAG
	F1spAR1-A	TCCCTATAAGAACGCTACAGAG
	F1spAF1-A	TGTCATGGCATGGCATGCAC
	GS2R6	ATGTTGTTGCCTCCTCGGAATG
	GS2F5	ACCCACAGGCCATATTCAAGG
	GS2R4	AGCCAGCTCCATTCCAGTCA
	GS2F2	TCGAGAGAATCACGGAGCAA
	WLR1	GGTCCTTCATACCTTCAGCG
promoter genomic PCR		
	GS2-A016 F1	ACAAACGGAATCTGACTTTGAAAAGTTTGCC
	GS2-A016 R1	CGCCGCTGCTTACTTACTTACTC
	GS2-B017 F1	CAAGGTCTGGTAATCCCATGTGTTGTGC
	GS2-B017 R1	CGCCGCTGCTTGCTTACTTACTTACTCC
	GS2-D018 F2	CCTGCTATTGCTTCGGATGA
	GS2-D018 R1	CGCCGCTACTTACTTACTTACTC
RT-PCR		
	mGS2-F2	TCCTCCCTCGTCTCGTCCGCGT
	mGS2-R2	AGTGCCCCGACGGAACCACAGG
TaGS2 mutants selection		
	In10F	TGGCTGCCACACAAATTACAG
	In10R	CCTTCTTGATCACGTCGAAAC
ChIP		
	qPCR-A-F1	TTGGTTTCTGGACCGGTTTG
	qPCR-A-R1	CAGTGGCATCTGGCTCTTGA
	qPCR-A-F2	GCGTGCTTGTCCGTCTCTCT
	qPCR-A-R2	ATCAGCGAATGAACGCAACA
	qPCR-A-F3	TGCAGGGTTGGAGGATCTG
	qPCR-A-R3	CTATCGAACGCGTACCCTTGA
	qPCR-A-F4	GCCCACAGGCCATATTCAAG
	qPCR-A-R4	AGATGAAAGGTACCAGGATGTTGTT
	qPCR-A-F5	TTTGCGAGTGAGAAACAACAATC
	qPCR-A-R5	ATCTTCGCGCATGCTCAGT
	qPCR-A-F6	ACGCCCTTCACCGACAAG
	qPCR-A-R6	GCGCCTGACGAGAAAACAG

	qPCR-B-F1	CGTCCCCGTCGTTGTCTCT
	qPCR-B-R1	ACGAACAAGCATTGCATCGA
	qPCR-B-F2	GGCTGGGCTGGGTATGG
	qPCR-B-R2	GGCAAAAAACCGTCCAAAGA
	qPCR-B-F3	TCAGAAGCAAATCAAGGGTACTCA
	qPCR-B-R3	GCAAACTATGCATGCAAGGAAA
	qPCR-B-F4	CCCGATAGAGCATCACCTTAATTT
	qPCR-B-R4	ACGCATGCTCAATGTGCTACAT
	qPCR-B-F5	CCCTTCACCGACAAGATCATC
	qPCR-B-R5	GGGTCCGCTGACGAGAAAA
	qPCR-B-F6	CATTTGGCCGTGACATTTCA
	qPCR-B-R6	TGATTTCAATTCCGGCGTAAAG
	qPCR-D-F1	CGTCCGCGTCCTTGTCA
	qPCR-D-R1	GAGAATCGAGGAAGCATTGCA
	qPCR-D-F2	GGCAGCCGCATCTTTCAT
	qPCR-D-R2	GCCCAGAATGCACACACATC
	qPCR-D-F3	ATCATCGCCGAGTACATCTGGTA
	qPCR-D-R3	GACGCGTGCGACGAGAA
	qPCR-D-F4	TCATCAAATATCGCCGTTTCC
	qPCR-D-R4	AGTGAACTGCTCATCCCGTAAAA
	qPCR-D-F5	CTGACCGAATGAGCATCCTTTT
	qPCR-D-R5	ACACCAGCTTGCTCCGTGAT
	qPCR-D-F6	GAACCTTTCACTTCGCCATGA
	qPCR-D-R6	TTCACTCGCCATACCCATGA
BSP		
	BSP-A-F1	TAGGTTATTAGTGTGGTTGTATTTGTATAAGAAG
	BSP-A-R1	TAACCCCRAAACCAATAATTA
	BSP-A-F2	ATGTTGTATTTGATAAAGGGGT
	BSP-A-R2	AATATTCCTTCCCTCTAACTCC
	BSP-A-F3	GTGTGTGTTTTTTTTGGGTAGA
	BSP-A-R3	TAATCAACCAAACCAAAACAAC
	BSP-A-F4	TGTTTTGGTTTGGTTGATTAGT
	BSP-A-R4	ATCAATCAATCTATAAAAACRAAAAT
	BSP-B-F1	TGTATTGTAGTYGGGTTATTGTAG
	BSP-B-R1	TCATCCCCTTTATCAAATACA
	BSP-B-F2	GTATTTGATAAAGGGGATGAAGG
	BSP-B-R2	CCAAAAAAAACACACACATCTC
	BSP-B-F3	GAGATGTGTGTGTTTTTTTTGG
	BSP-B-R3	AACCAAAACACAAAAAAACAAAC
	BSP-B-F4	GTTTTTTTGTGTTTTGGTTGATTAG
	BSP-B-R4	CAATCAATCCATAAAAACRAAC
	BSP-D-F1	GTTTATGTGTGTTGTTGTTGGG
	BSP-D-R1	CAAACCCAACCAAATACAACA

BSP-D-F2	TGTTGTATTTGGTTGGGTT
BSP-D-R2	AATCRCRCTAAAAATATTTATC
BSP-D-F3	ATATTTGATAAAGGGGATGAAGG
BSP-D-R3	CAATATATTCCTTATTCCTTCTCTCC
BSP-D-F4	GGAGAGAAGGAATAAGGAATATATTG
BSP-D-R4	CAAAACAAAAAAAACAAAACCA
BSP-D-F5	TTTTTTGTTGAAGGTTTGGAG
BSP-D-R5	TAAAAATCAATCCATCAATCCA
**TaqMan absolute quantification real-time RT-PCR**	
tubb2-F	ATGGTTCTTGACAACGAGGCT
tubb2-R	AGGCGAGGGAATGGGATAA
tubb2-P	ATCTGTTTCAGGACTCTTAAGCTGACCACAC
TaGS2A-F	ACAAGATCATCGCCGAGTACATCTG
TaGS2A-R	ACTGTCTTCTCCAGGAGCCTGCCCG
TaGS2A-P	CCCGTCAGAGCTACCCAAATGGAATTATGAC
TaGS2B-F	ACAAGATCATCGCCGAGTACATCTG
TaGS2B-R	ACTGTCTTCTCCAGGAGCCTGGCCT
TaGS2B-P	CCCGTCAGAGCTACCGAAATGGAACTACGAT
TaGS2D-F	AGTACATCTGGGTTGGAGGATCTGG
TaGS2D-R	TGTGCTCGATCCGTCGTAGTT
TaGS2D-P	CCTCAGGAGCAAATCAAGGACGATTTCAAAGCC
**SYBR relative quantification real-time RT-PCR**	
qGS2-F1	CCGAGACCACGATCCTGT
qGS2-R1	GGTCCTTCATACCTTCAGCG
**Enzyme accessibility assay**	
qGAPDH-F	TTAGACTTGCGAAGCCAGCA
qGAPDH-R	AAATGCCCTTGAGGTTTCCC

*R：A or G；Y：C or T
